# 

**Published:** 1875-08

**Authors:** 


### Books and Pamphlets Received.

Cyclopædia of the Practice of Medicine. Edited by Dr. H von Ziemssen. Vol. X. Diseases of the Female Sexual Organs. By. Prof. Carl Schrœder, of Erlangen. American Translation. Edited by Albert H. Buck, M. D. New York: Wm. Wood & Co., 1875.

Plain Directions for the Care of the Sick, and Receipts for Sick People. Plain Directions for Accidents, Emergencies and Poisons.

First Annual Report of the St. Catherines Training School, and Nurses' Home, in Connection with the General and Marine Hospital.

Fourth Annual Announcement of the College of Medicine of Syracuse University, Syracuse, N. Y.

